# Assessing mRNA translation in mouse adult microglia and bone-marrow-derived macrophages

**DOI:** 10.1016/j.xpro.2023.102559

**Published:** 2023-09-14

**Authors:** Ignazio Antignano, Lily Keane, Melania Capasso

**Affiliations:** 1 Deutsches Zentrum für Neurodegenerative Erkrankungen (DZNE), 53127 Bonn, Germany

**Keywords:** Flow Cytometry/Mass Cytometry, Immunology, Neuroscience

## Abstract

Protein synthesis, or mRNA translation, is the biological process through which genetic information stored in messenger RNAs is encoded into proteins. Here, we present an optimized protocol for assessing the translation rate in mouse adult microglia and cultured bone-marrow-derived macrophages. We describe steps for isolating cells, treating them with a puromycin-analog probe, and fluorescently labeling the puromycylated-polypeptide chains. We then detail their quantification by flow cytometry or with a fluorescent plate reader.

For complete details on the use and execution of this protocol, please refer to Keane et al. (2021).^1^

## Highlights

• A fast and easy protocol to assess translation rate in *ex vivo* microglia and BMDMs
• Fluorescent readout measured by flow cytometry or a plate reader
• Flow cytometry option allows simultaneous detection of surface or intracellular markers
• Avoids use of radioactive tracers

## Abstract (editor highlights)

Publisher’s note: Undertaking any experimental protocol requires adherence to local institutional guidelines for laboratory safety and ethics.

## Graphical abstract

**Figure:**
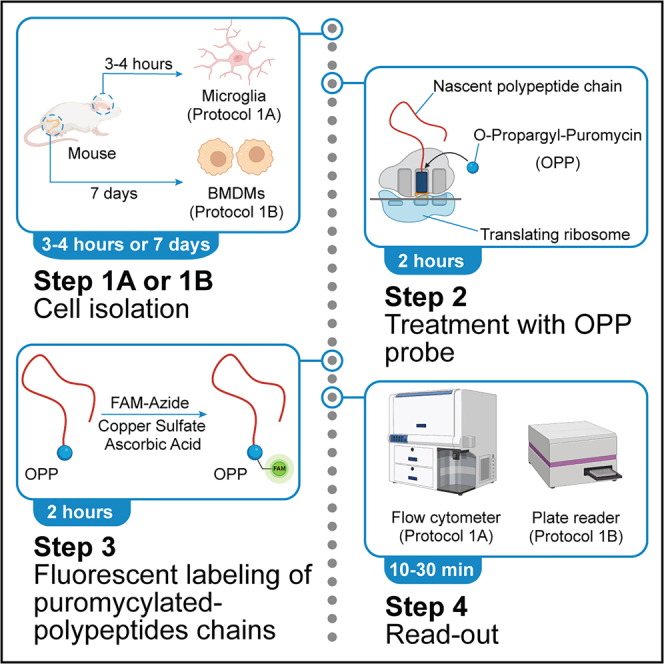

## Before you begin

Protein synthesis consists in translating the genetic information stored in mRNAs into proteins. This multi-step process, from ribosome loading onto mRNAs to protein synthesis and folding is tightly regulated by post-transcriptional regulatory mechanisms, allowing cells to respond to environmental cues.^2^

Macrophages and microglia are extremely plastic cells and heavily rely on protein synthesis in order to rapidly produce inflammatory mediators in response to different stimuli.^1^^,^^3^^,^^4^ This article describes an easy-to-use protocol in order to assess the translation rate of myeloid cells and represents a safer alternative to methods based on the incorporation of radioactive tracers, such as S35 methionine, into nascent polypeptide chains. The Protein Synthesis Assay Kit (Cayman Chemical, Cat. No. 601100) contains O-Propargyl-Puromycin (OPP), an alkyne analog of puromycin, which is incorporated into nascent polypeptide chains and terminates translation. The kit also contains a 5-Carboxyfluorescein (5-FAM)-Azide, a fluorescent dye that can be conjugated to OPP by copper(I)-catalyzed azide-alkyne cycloaddition. The excitation and emission of 5-FAM are respectively 485 nm and 525 nm, and it can be detected in the commonly used settings configured for FITC (fluorescein isothiocyanate) or GFP (green fluorescent protein) in a flow cytometer or plate reader.

Prior to starting the execution of this protocol, read carefully the sections below describing the steps for cell isolation. Prepare in advance all buffers required, which are described in sections “key resources table” and “materials and equipment”. For adult microglia, brain cells are isolated from mouse brains using the Adult Brain Dissociation protocol from Miltenyi Biotec. In the subsequent flow cytometry analysis, microglia are identified by staining of the surface markers CD11b and CD45, microglia are CD11b^high^ and CD45^int^.^5^^,^^6^ For disease-related applications, purinergic receptor 12 (P2ry12) can be included to distinguish between microglia and infiltrating monocytes. For bone marrow-derived macrophage (BMDM) cultures, the growth factor M-Csf (Macrophage colony-stimulating factor, or Csf-1), which promotes monocyte differentiation into macrophages, is required. This can be obtained by supplementing the culture media with supernatant from L929 cells, which naturally produce M-Csf.^7^ The L929 supernatant needs to be prepared prior to starting bone marrow isolation. The protocol used for L929 supernatant production is described in “materials and equipment”.

Finally, we have not previously validated this assay in other cell types, nonetheless, this protocol might represent a valuable starting point to assess translation rate in other cells of interest.

### Institutional permissions

Mice were housed according to German NRW Landesamt für Natur, Umwelt und Verbraucherschutz Nordrhein-Westfalen (LANUV NRW) guidelines. All experiments involving mice were conducted in accordance with German LANUV NRW license 81-02.04-2018.A215.

### Isolation of brain cells from adult mice

**Timing: 3–4 h**

This section describes the isolation of mouse brain cells, which will be used to assess translation in adult microglia.

1. If required, treat mice according to your experimental protocol (i.e., intraperitoneal injection of 5 mg/kg LPS for 4 h).
2. Sacrifice animals according to your institutional permissions.

***Optional:*** Mice can be perfused before brain cell isolation to remove peripheral blood cells, nonetheless, this step is not essential, since it is possible to gate on microglia in the flow cytometry analysis.

3. Remove brain and process it according to the Adult Brain Dissociation protocol from Miltenyi Biotec (https://www.miltenyibiotec.com/DE-en/products/adult-brain-dissociation-kit-mouse-and-rat.html#130-107-677). Troubleshooting 4 and troubleshooting 5. Ensure to remove debris using the debris removal step according to manufacturer’s guidelines but skip the Red Blood Cell Lysis step.

***Note:*** we do not perform the Red Blood Cell Lysis step, since in our experience, this can reduce yield and viability of our cells of interest. If mice are not perfused, red blood cells remain in the cell suspension; they do not interfere with the experimental protocol and are then gated out in the flow cytometry analysis.

***Note:*** Cells isolated from only one of the two brain hemispheres will be sufficient for the assay.

4. Count cells using an automated cell counter or a hemocytometer.
5. Adjust brain cell suspension to 2 × 10^6^ cells/100 μL in microglia medium (MM).
6. Proceed to determine translation rate as described below in the flow cytometry-based translation assay in adult microglia paragraph – step 1.

### Bone marrow-derived macrophages culture protocol

**Timing: 7 days**

The following describes the isolation and cell differentiation of bone marrow-derived macrophages (BMDMs) generated according to the protocol by Joachim Weischenfeldt and Bo Porse,^8^ with minor changes, as described below. The L929-derived conditioned medium used for bone marrow cell differentiation into macrophages is obtained by harvesting the supernatant of L929 cells, which secrete the growth factor M-Csf^7^ and should be prepared in advance as described in “materials and equipment”.

1. Sacrifice mouse and collect femur and tibia as described.^1^^,^^8^
2. Under a laminar flow hood, flush the bones with HBSS using a 5-mL syringe and a 27-gauge needle.
3. Strain bone marrow (BM) cells through a 70 μm cell strainer into a 50 mL tube.
4. Wash the strainer with 5 mL of HBSS.
5. Spin down at 300 × *g* for 10 min at 18°C–24°C and carefully remove the supernatant.
6. Resuspend BM cells by gently pipetting up and down to get a single-cell suspension in HBSS.
7. Count BM cells using an automated cell counter or hemocytometer.
8. Adjust BM cell suspension to 12 × 10^6^ cells/mL in BM medium (BMM).
9. Add 1 mL of cell suspension to a 150 mm Petri dish containing 15 mL of BMM and 4 mL of L929-conditioned medium (L929CM) to get a total volume of 20 mL per dish.
10. Gently tilt the plate backward and forward to obtain a homogenous cell suspension covering the entire dish (do not rotate the dish).

***Note:*** the final amount of L929CM in each plate is 20% (v/v).

11. Differentiate BM cells in a cell culture incubator at 37°C/5% CO_2_ for 6 days.
12. On day 4, carefully remove half of the medium (∼10 mL) with a pipette and carefully add a mixture of 8 mL of BMM and 2 mL of L929CM per dish.
13. On day 6, carefully wash the dish twice with pre-warmed DPBS (without calcium, magnesium) to remove dead cells and debris.
14. Incubate cells in pre-warmed dissociation buffer (DB) for 10 min in a cell culture incubator at 37°C/5% CO_2_. Check if cells have detached using a microscope, if not, continue to incubate cells at 37°C/5% CO_2_ until they do.
15. Gently scrape the plate with a cell scraper.
16. Collect cell suspension into a 50 mL tube with a 10 mL pipette.
17. Wash cell culture dish with 5 mL of pre-warmed DPBS (without calcium, magnesium).
18. Scrape again.
19. Collect this second cell suspension (from step 17–18) with a 10 mL pipette and combine it with the first collection already in the 50 mL tube from step 16.
20. Spin down at 300 × *g* for 10 min at 18°C–24°C and remove supernatant.
21. Resuspend BMDMs by gently pipetting up and down to get a single-cell suspension.
22. Count BMDMs using an automated cell counter or a hemocytometer.
23. Adjust cell suspension to 50,000 cells/100 μL in BMM

***Note:*** No L929CM is added to BMDMs from now on.

24. Plate 50,000 cells per well for at least two or three technical replicates per condition.
25. Allow cells to adhere in a cell culture incubator at 37°C/5% CO_2_ for 12–16 h.
26. On the following day (day 7), treat BMDMs according to the experimental design (i.e., stimulate BMDMs with 100 ng/mL LPS for 3 h).
27. Proceed to determine translation rate as described below in the in-vitro translation assay for cultured bone marrow-derived macrophages (BMDMs) paragraph – step 1.

## Key resources table

**Table:** 

REAGENT or RESOURCE	SOURCE	IDENTIFIER
**Antibodies (for FACS analysis)**		

Anti-CD11b-BUV395 (clone: M1/70) – dilution 1:200	BD Biosciences	Cat#563553
Anti-CD45-BV711 (clone: 30-F11) – dilution 1:200	BioLegend	Cat#103147
LIVE/DEAD Fixable Near-IR for 633 or 635 nm excitation – dilution 1:1000	Thermo Fisher Scientific	Cat#L10119
TruStain FcX (anti-mouse CD16/32) antibody (clone: 93) – dilution 1:200	BioLegend	Cat#101320

**Biological samples**		

Brain cells (including microglia)	In this protocol	N/A
Bone-marrow-derived macrophages (BMDMs)	In this protocol	N/A

**Chemicals, peptides, and recombinant proteins**		

Cell culture water	Sigma-Aldrich	Cat#W3500-500ML
DPBS (with calcium, magnesium, glucose, pyruvate)	Thermo Fisher Scientific	Cat#14287080
DPBS (without calcium, magnesium)	Sigma-Aldrich	Cat#D8537
Fetal bovine serum (FBS)	PAN-Biotech	Cat#P30-193306
Dulbecco′s modified Eagle′s medium-high glucose (DMEM)	Sigma-Aldrich	Cat#D5671
Sodium pyruvate (100 mM)	Thermo Fisher Scientific	Cat#11360039
Penicillin-Streptomycin (10.000 U/mL)	Thermo Fisher Scientific	Cat#15140122
GlutaMAX supplement	Thermo Fisher Scientific	Cat#35050087
EDTA, 0.5 M sterile solution, pH 8.0	VWR	Cat#E177-100ML
HEPES 1 M sterile	Thermo Fisher Scientific	Cat#15630056
HBSS, without calcium, magnesium, phenol red	Thermo Fisher Scientific	Cat#14175053
Cell Dissociation Buffer, enzyme-free	Thermo Fisher Scientific	Cat#13151014
Trypsin (2.5%), without phenol red	Thermo Fisher Scientific	Cat#15090046
Paraformaldehyde (PFA)	Sigma-Aldrich	Cat#158127
4% PFA/PBS	In-house	N/A
70% Ethanol	Carl Roth	Cat#T913.3
Lipopolysaccharides from *Escherichia coli* O111:B4 (LPS)	Sigma-Aldrich	Cat#L3024-10MG

**Critical commercial assays**		

Protein Synthesis Assay	Cayman Chemical	Cat#601100
Adult Brain Dissociation Kit, mouse and rat	Miltenyi Biotec	Cat#130-107-677

**Experimental models: Cell lines**		

L929 cell line [lot: 17110076 (07/2018)]	Sigma-Aldrich	Cat#85011425-RNA-5UG

**Experimental models: Organisms/strains**		

C57BL/6J maintained in individually ventilated cages (IVCs); age- and sex-matched, co-housed	In-house	N/A

**Software and algorithms**		

FlowJo software (v10)	BD Biosciences	https://www.flowjo.com/solutions/flowjo
Prism (v9.5.1)	GraphPad by Dotmatics	https://www.graphpad.com/scientific-software/prism/

**Other**		

150 mm × 15 mm not TC-treated sterile Petri dish	Corning	Cat#351058
75 cm^2^ cell culture flask	Greiner Bio-One	Cat#658175
96-well clear bottom black TC-treated sterile microplate	Corning	Cat#3603
96-well clear V-bottom sterile microplate	Greiner Bio-One	Cat#651180
Cell strainer, pore size 70 μm	Sarstedt	Cat#83.3945.070
5 mL syringes	Braun	Cat#4606051V
Needles-27G 1/2″	BD	Cat#300635
Cell scraper	Greiner Bio-One	Cat#541070
5 mL round bottom polystyrene tubes (FACS tubes)	Corning	Cat#352054
Nalgene Rapid-Flow sterile vacuum filter units (0.2 μm pore size)	Thermo Fisher Scientific	Cat#567-0020
50 mL tubes	Greiner Bio-One	Cat#227-261
15 mL tubes	Greiner Bio-One	Cat#188-271
FACSymphony A5 – flow cytometer	BD Biosciences	N/A
Cell culture incubator at 37°C/5% CO_2_	Memmert	N/A
Plate centrifuge	Thermo Fisher Scientific	N/A

## Materials and equipment

Materials relative to the Protein Synthesis Assay described below can also be found in the corresponding assay protocol (https://www.caymanchem.com/product/601100/protein-synthesis-assay-kit). Since we have slightly modified the manufacturer’s protocol, in order to evaluate the translation rate of adult microglia cells from multiple samples using a V-bottom plate instead of single tubes, we report the preparation of materials specific to our assay.

The equipment needed for the execution of this assay are described in the section “Others” in the key resources table.

### Microglia medium (MM)

• Prepare a solution containing DPBS (with calcium, magnesium, glucose, and pyruvate) and 0.5% fetal bovine serum (FBS). Store it up to 2 months at 4°C and pre-warmed in a water bath at 37°C prior to using it.

**Table:** Bone marrow medium (BMM)

Reagent	Final concentration	Amount
DMEM	N/A	435 mL
Fetal bovine serum (FBS)	10% (v/v)	50 mL
Sodium Pyruvate (100 mM)	1 mM	5 mL
Penicillin-Streptomycin (10.000 U/mL)	1% (v/v)	5 mL
GlutaMAX Supplement (100X)	1X	5 mL
**Total**	**N/A**	**500 mL**

Store at 4°C and up to 2 months, pre-warm in a water bath at 37°C prior to use.

**Table:** L929 medium (L929M), only for BMDMs

Reagent	Final concentration	Amount
DMEM	N/A	430 mL
Fetal bovine serum (FBS)	10% (v/v)	50 mL
Sodium Pyruvate (100 mM)	1 mM	5 mL
Penicillin-Streptomycin (10.000 U/mL)	1% (v/v)	5 mL
GlutaMAX Supplement (100X)	1X	5 mL
HEPES	1%	5 mL
**Total**	**N/A**	**500 mL**

Store it up to 2 months at 4°C, pre-warm in a water bath at 37°C prior to use.

### 0.25% Trypsin in PBS, only for BMDMs

• Dilute 2.5% Trypsin in PBS (without calcium, magnesium) in 1:10 ratio (e.g., 1 mL of 2.5% Trypsin in 9 mL PBS).
• Mix by vortexing, aliquot in 15 mL tubes and store at –20°C.
• Thaw the solution when needed in a water bath at 37°C. Use within maximum one day after thawing.

### L929-conditioned medium (L929CM), only for BMDMs

The L929-conditioned medium can be generated according to the protocol by Joachim Weischenfeldt and Bo Porse,^8^ with minor changes, described below.

• Seed 0.47 × 10^6^ L929 cells in a 75 cm^2^ flask containing L929M (final volume 55 mL) and grow cells for 7 days in a 37°C/5% CO_2_ incubator.
• At day 7, collect supernatant, filter with a 0.2 μm pore filter and prepare aliquots for freezing.
• Store frozen aliquots at –80°C. When needed, thaw aliquot in a water bath at 37°C and use within 1 day.

### Dissociation buffer (DB), only for BMDMs

• Dilute Cell Dissociation Buffer in pre-warmed DPBS (without calcium, magnesium) at 1:1 ratio.
• Prepare enough dissociation buffer for the total amount of plates to be harvested. Once diluted for the amount needed, Cell Dissociation Buffer has to be used within one day.

### Assay Buffer (1X)

• Dilute the Cell-Based Assay TBS (10X) contained in the assay kit with 90 mL of cell culture water. Mix well at 18°C–24°C.
• The diluted Assay Buffer should be stable for at least one year when stored at 18°C–24°C.

### Cycloheximide solution, only for adult microglia

• Prepare a solution containing 100 μg Cycloheximide in 100 μL of microglia medium (MM) by diluting 1:500 a stock solution of 50 mg/mL for each sample.
• Prepare enough solution based on the number of samples to be pre-treated with Cycloheximide.
• Vortex and keep it at 18°C–24°C.

***Note:*** Cycloheximide is dissolved in DMSO and stored at –20°C. Keep the vial at 18°C–24°C for at least 30 min and vortex prior to using it.

### O-Propargyl-Puromycin (OPP) stock solution, only for adult microglia

• The following amount refers to a total well volume of 110 μL per sample, which includes the 10 μL of OPP stock solution that needs to be added to each of the indicated conditions.
• Multiply the following reagents described in the table by the total number of conditions and samples according to the experimental design.

**Table:** 

Reagent	Dilution factor	Amount
OPP stock solution	1:400	0.275 μL
Cell culture medium	-	9.725 μL
**Total**	**N/A**	**10 μL**

Prepare the solution in advance to rapidly add to each well. The solution can be stored at 18°C–24°C but it needs to be used immediately.

**Table:** O-Propargyl-Puromycin (OPP) working solution, only for BMDMs

Reagent	Dilution factor	Amount
OPP stock solution	1:400	0.25 μL
Cell culture medium	-	99.75 μL
**Total**	**N/A**	**100 μL**

Prepare the solution in advance to rapidly add it to each well. The solution can be stored at 18°C–24°C and it needs to be used immediately.

**Table:** Cycloheximide + O-Propargyl-Puromycin (CHX+OPP) working solution, only for BMDMs

Reagent	Dilution factor	Amount
OPP stock solution	1:400	0.25 μL
Cycloheximide	1:500	0.20 μL
Cell culture medium	-	99.55 μL
**Total**	**N/A**	**100 μL**

Prepare the solution in advance to rapidly add it to each well. The solution can be stored at 18°C–24°C and needs to be used immediately.

**Table:** FACS Buffer, only for adult microglia

Reagent	Dilution factor	Amount
DPBS (without calcium, magnesium)	-	494.5 mL
EDTA	2 mM	2 mL
Fetal bovine serum	0.5% (v/v)	2.5 mL
**Total**	**N/A**	**500 mL**

Mix the solution avoiding generating air bubbles. Prepare the solution in advance and store at 4°C up to 2 months. FACS Buffer must be ice-cold during the preparation of the FACS staining stock solution.

### FACS staining stock solution, only for adult microglia

• The following amount refers to a total well volume of 120 μL per one sample, which includes the 10 μL of FACS stock solution that needs to be added to each of them.
• Multiply the following reagents described in the table by the total number of samples/conditions according to the experimental design.

**Table:** 

Reagent	Dilution factor	Amount
anti-CD11b - BUV395 (clone: M1/70)	1:200	0.6 μL
anti-CD45 - BV711 (clone: 30-F11)	1:200	0.6 μL
LIVE/DEAD Fixable Near-IR for 633 or 635 nm excitation	1:1000	0.12 μL
TruStain FcX (anti-mouse CD16/32) Antibody (clone: 93)	1:200	0.6 μL
FACS Buffer	-	8.08 μL
**Total**	**N/A**	10 μL

Prepare the solution immediately before use, protect it from light and keep on ice.

***Note:*** We incubate FcR block (TruStain FcX) together with primary antibodies against CD11b and CD45. This strategy, designed to reduce incubation times, works with these antibodies but should be tested if users utilize different ones.

### Cell-based assay 5 FAM-Azide Staining Solution

• The following amount refers to a single condition for one sample.
• Multiply the following reagents described in the table by the total number of conditions and samples according to the experimental design.

**Table:** 

Reagent	Dilution factor	Amount
Cell-Based Assay 5 FAM-Azide Stock Solution	1:1000	0.1 μL
Copper Sulfate Solution	1:1000	0.1 μL
Ascorbic Acid Solution	1:100	1 μL
Cell-Based Assay Buffer (1X)	-	98.8 μL
**Total**	**N/A**	100 μL

Prepare the solution immediately before use, protected from light and store at 18°C–24°C.

## Step-by-step method details

**Figure 1:**
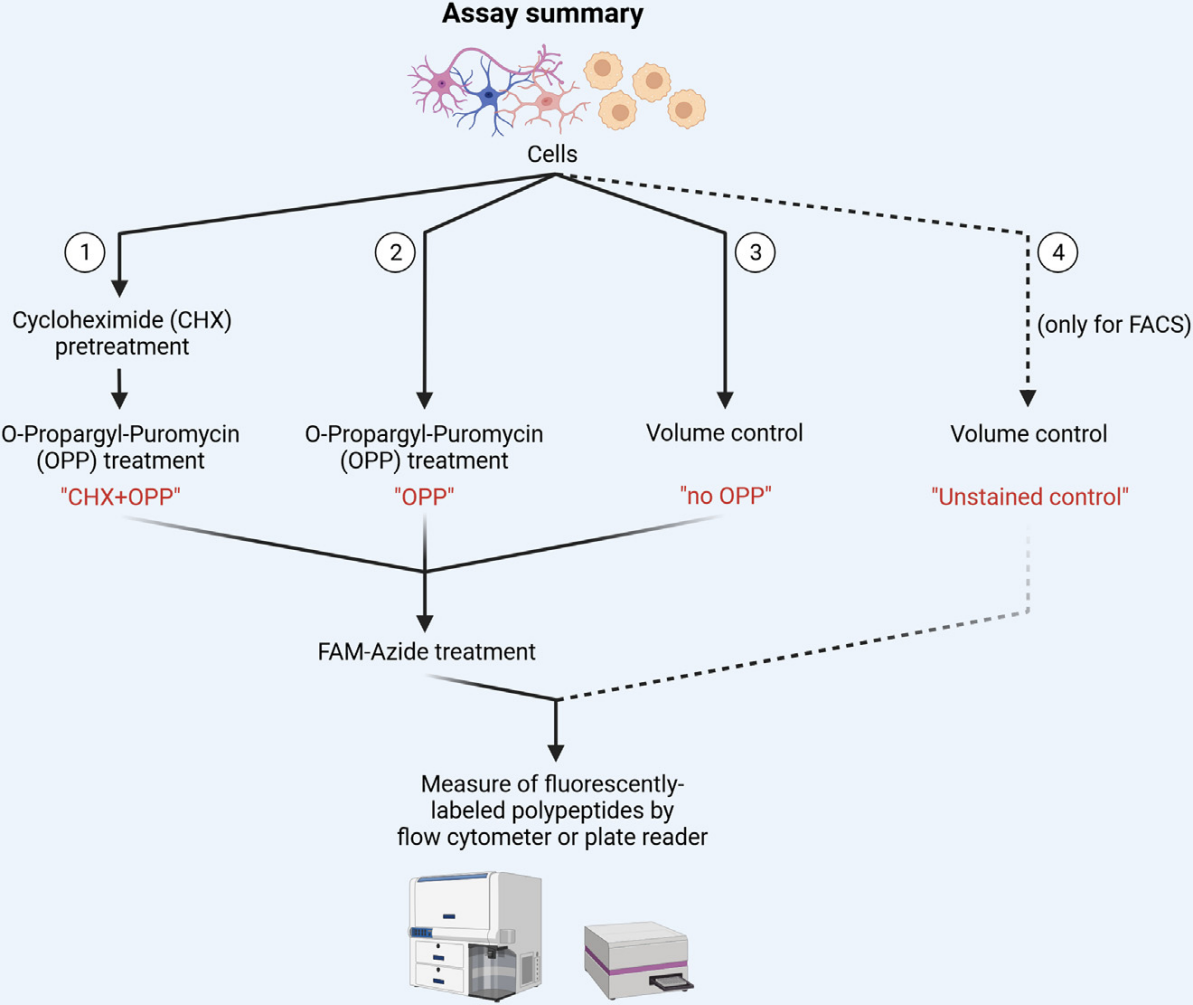
Visual summary of steps required in the protein synthesis assay Image created with BioRender.com.

Each sample will require the following conditions: 1) cells treated with Cycloheximide (CHX) and OPP (hereafter defined as “CHX+OPP”) as a control for reduced translation; 2) cells treated with OPP (hereafter defined as “OPP”); 3) cells not treated with OPP but subsequently treated with FAM-Azide (defined as “no OPP”); 4) cells used as “unstained control” and “live/dead control”, only for FACS analysis (Figure 1).

***Note:*** If the number of cells is limited, conditions 3) and 4) can be obtained by mixing cells from multiple samples.

### Flow cytometry-based translation assay in adult microglia

**Timing: 5 h**

The following protocol describes the steps needed for determining the translation rate in adult mouse brain cells. Previous steps 1–6 were described in section “before you begin”, isolation of brain cells from adult mice.

1. Plate 100 μL of brain cell suspension per condition in a V-bottom plate.
2. Spin down the plate at 400 × *g* for 5 min at 18°C–24°C and carefully aspirate the supernatant and immediately proceed to next step. Troubleshooting 3.
3. Cycloheximide (CHX) pre-treatment, only for CHX+OPP control sample
  a. Add 100 μL of Cycloheximide solution to CHX+OPP samples (control for reduced translation). Mix well and avoid generating bubbles when pipetting up and down with a P200 pipette.
  b. Add 100 μL of Microglia medium (MM) to all other samples and resuspend with a P200 pipette.
  c. Incubate cells in a cell culture incubator at 37°C for 15 min. After treatment, proceed immediately to point 4.
4. O-Propargyl-Puromycin (OPP) incubation
  a. Add 10 μL of OPP stock solution only to samples designated as “OPP” and “CHX+OPP”. Mix well by pipetting up and down without generating bubbles.
  b. To the remaining samples, add 10 μL of media. Incubate cells in a cell culture incubator at 37°C for 1 h. Each sample now has a final volume of 110 μL.

***Note:*** the FACS staining stock solution can be prepared 10–15 min before the end of the OPP treatment.

5. Surface marker staining.
  a. Add 10 μL of the previously prepared FACS staining stock solution to each condition (no OPP, OPP and OPP + CHX) but not the unstained controls.
  b. Mix to get a single cell suspension by gently pipetting without generating bubbles.
  c. For the unstained control well, use 10 μL of MM only and for the live/dead control, use 10 μL of MM containing only the live/dead staining dye.
  d. Protect plate from light and incubate for 10 min at 37°C.
  e. Spin down the plate at 400 × *g* for 5 min at RT and carefully aspirate the supernatant.

***Note:*** If channel compensation on the flow cytometer is not performed with beads, samples with single stains and Fluorescent Minus One (FMO) stains will be required.

**CRITICAL:** Fluorescently labeled antibodies used for FACS analysis are light-sensitive. Therefore, it is advisable to perform the following steps shielding samples from light. In order to achieve this, cover the plate with parafilm (to avoid spillages in neighboring wells) or a 96-well plate lid; then cover the plate with aluminum foil.

**CRITICAL:** Aspirating supernatant with a vacuum pump should be avoided in order to reduce the risk of aspirating cells. This step can be carried out with a pipette, using a multichannel pipette if working with a considerable number of samples. The same applies to the subsequent steps.

6. Cell Fixation.
  a. Resuspend cells in 100 μL of 4% PFA/PBS per well. Mix gently to ensure a single cell suspension. Protect plate from light and incubate at 18°C–24°C for 5 min. Troubleshooting 1.
  b. Spin down the plate at 400 × *g* for 5 min at 18°C–24°C and carefully aspirate the supernatant.
7. Washing cells with Cell-Based Assay Wash Buffer.
  a. Resuspend cells in 200 μL of Cell-Based Assay Wash Buffer provided with the kit. Mix to ensure a single cell suspension and incubate the plate protected by light at RT for 5 min.
  b. Spin down the plate at 400 × *g* for 5 min at 18°C–24°C and carefully aspirate the supernatant.
  c. Repeat cell washes steps (7a and 7b) for a total of three times.

***Note:*** Cell-Based Assay Wash Buffer contains 0.1% of Polysorbate 20 (also known as Tween 20), a nonionic surfactant required for cell permeabilization,^9^ in order to allow FAM-Azide to enter cells and encounter OPP-conjugated nascent peptides.

**Figure 2:**
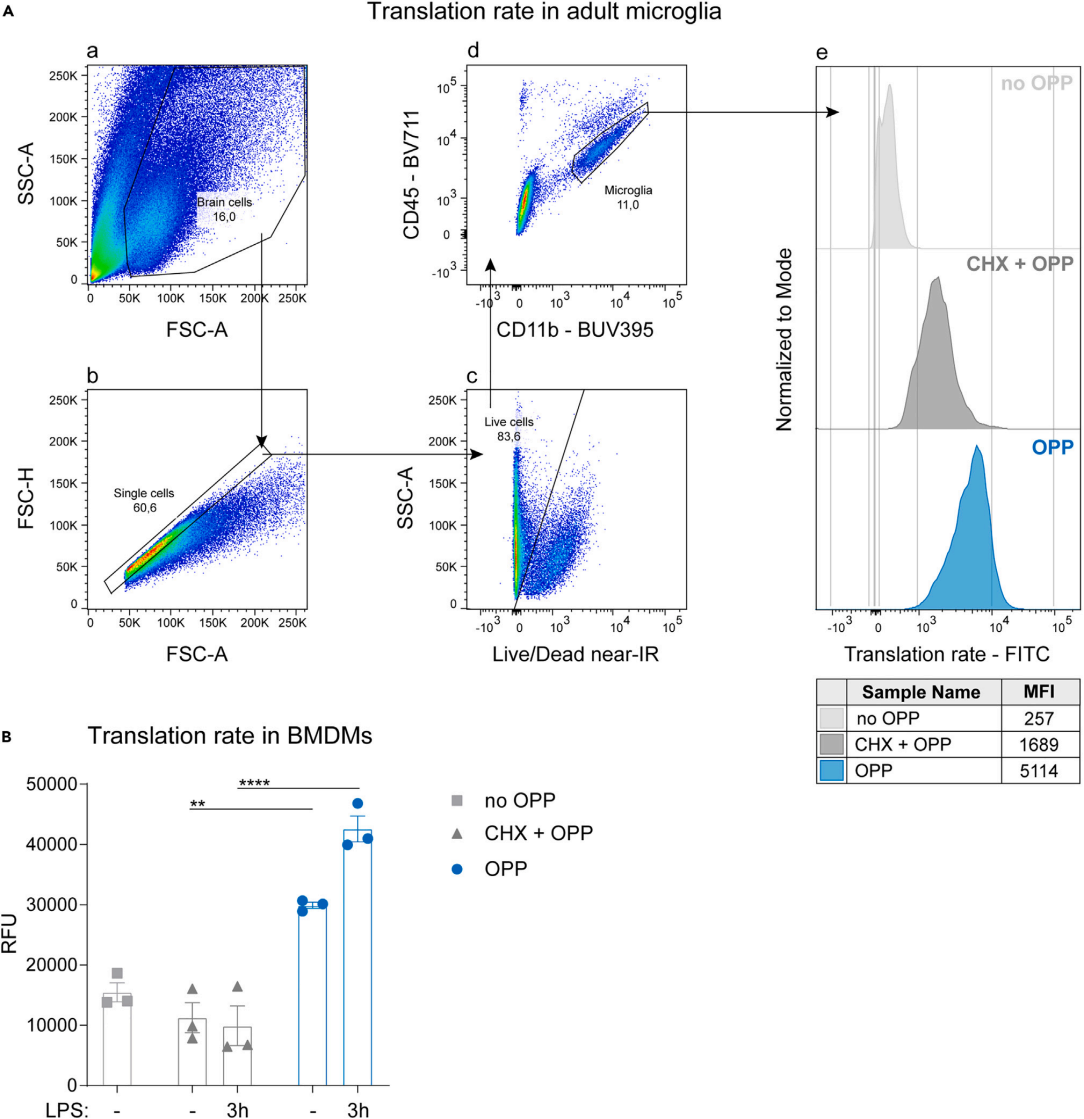
Expected outcomes of the protein synthesis assay in untreated (no OPP), Cycloheximide- and OPP-treated (CHX+OPP), OPP-treated (OPP) adult microglia or BMDMs (A) Gating strategy applied for the identification of adult microglia cells: a) Forward scatter (FSC-A) – Side scatter (SSC-A) plot of cells contained in brain cell suspension; b) FSC-H – FSC-A plot to identify single cells; c) SSC-A – LIVE/DEAD plot to identify viable cells (=negative cells) ; d) dot plot for CD11b and CD45 channels, microglia are identified based on their high expression of CD11b and intermediate expression of CD45; e) histograms for 5-FAM channel; top panel: cells with no OPP and therefore no staining; middle panel: cells with OPP but treated with CHX, showing reduced translation when compared with cells not treated with CHX (bottom panel); bottom panel: cells treated with OPP showing full translation rate. Median Fluorescence Intensities (MFI) of no OPP, CHX+OPP and OPP are reported in in the table below the histograms. (B) Relative fluorescence intensity (RFU) of the protein synthesis rate in wild-type BMDMs at steady state and after stimulation with 100 ng/mL LPS for 3 h and assessed with a fluorescent plate reader. Data adjusted from Keane et al.^1^ and displayed as mean ± SEM (n = 3). *P* adjusted value (*P*): ∗∗*P* < 0.01, ∗∗∗∗*P* < 0.0001, 2-way ANOVA with Tukey correction for multiple comparisons.

8. FAM-Azide staining.
  ***Note:*** During the last centrifugation steps (7a-c), start preparing the 5 FAM-Azide staining solution in a tube, protected from light.
  **CRITICAL:** FAM-Azide is light sensitive. Carry out the following steps without direct exposure to light.
  a. Gently resuspend cells in 100 μL of 5 FAM-Azide Staining Solution in order to obtain a single cell suspension. Incubate cells for 30 min in the dark at 18°C–24°C.
  b. Spin down the plate at 400 × *g* for 5 min at 18°C–24°C and carefully aspirate the supernatant.
  c. Resuspend cells in 200 μL of Cell-Based Assay Wash Buffer provided with the kit. Mix gently to ensure a single cell suspension and incubate the plate protected from light at 18°C–24°C for 5 min.
  d. Spin down the plate at 400 × *g* for 5 min at 18°C–24°C and carefully aspirate the supernatant.
  e. Repeat washing steps (8c and 8d) for a total of three times.
  f. Resuspend cells in 200 μL of previously prepared Assay Buffer (1X). Mix well by pipetting up and down to get a single cell suspension. Transfer the sample to a FACS tube and proceed immediately to analyze the sample by Flow cytometry. An example of the gating strategy is reported in Figure 2. Troubleshooting 2.
    ***Optional:*** To maximize cell recovery in step 8f, resuspend cells in 100 μL of Assay Buffer (1X); transfer sample to the corresponding FACS tube and keep the tube on ice and in the dark. Then use an extra 100 μL of Assay Buffer to further wash the well, collecting any remaining cell and transferring it into the corresponding sample tube.

### In-vitro translation assay for cultured bone marrow-derived macrophages (BMDMs)

**Timing: > 5 h**

This section describes the steps needed for determining the translation rate in differentiated BMDMs. Previous steps 1–26 were described in section “before you begin”, bone marrow-derived macrophages culture protocol.

1. Spin down the plate with BMDMs at 400 × *g* for 5 min at 18°C–24°C, carefully aspirate the supernatant and proceed immediately to the next step. Troubleshooting 3.

***Note:*** Although BMDMs are strongly adherent cells, we follow the Protein Synthesis Assay manufacturer’s recommendation to centrifuge the plate, as described in the assay protocol related to plate reader detection.

***Note:*** if working with a considerable number of samples, the supernatant can be removed more quickly by using a multichannel pipette. The same applies to the subsequent steps.

2. O-Propargyl-Puromycin (OPP) incubation with or without Cycloheximide.
  a. Add 100 μL of DMEM to the negative control (“no OPP”), 100 μL of OPP working solution (“OPP”) and 100 μL OPP working solution with cycloheximide (“CHX+OPP”), previously prepared, to the respective cell-containing wells.
    ***Note:*** For BMDMs, no pre-treatment with Cycloheximide with our time point of 3 h was required.
  b. Incubate cells in a cell culture incubator at 37°C/5% CO_2_ for 2 h.
  c. After treatment, spin down the plate at 400 × *g* for 5 min at 18°C–24°C and carefully aspirate the supernatant. Proceed immediately to step 30.
3. Cell Fixation.
  a. Add 100 μL of 4% PFA/PBS to each well and incubate at 18°C–24°C for 5 min.
  b. Spin down the plate at 400 × *g* for 5 min at 18°C–24°C and carefully aspirate the supernatant. Troubleshooting 1.
4. Washing cells with Cell-Based Assay Wash Buffer.
  a. Add 100 μL of Cell-Based Assay Wash Buffer provided with the kit and incubate the cells at 18°C–24°C for 5 min.
  b. Spin down the plate at 400 × *g* for 5 min at 18°C–24°C and carefully aspirate the supernatant.
  c. Repeat washing steps (4a and 4b) for a total of three times.

***Note:*** Cell-Based Assay Wash Buffer contains 0.1% of Polysorbate 20 (also known as Tween 20), a nonionic surfactant required for cell permeabilization,^9^ in order to allow FAM-Azide to enter cells and encounter OPP-conjugated nascent peptides.

***Note:*** During the last centrifugation steps (4a-c), start preparing the 5 FAM-Azide staining solution in a tube protected by light.

**CRITICAL:** FAM-Azide is light sensitive. All the next steps must be performed without direct exposure to light. Perform incubations in the dark.

5. FAM-Azide staining.
  a. Add 100 μL of 5 FAM-Azide Staining Solution and incubate cells for 30 min in the dark at 18°C–24°C.
  b. Spin down the plate at 400 × *g* for 5 min at 18°C–24°C and carefully aspirate the supernatant.
  c. Add 100 μL of Cell-Based Assay Wash Buffer provided with the kit and incubate the plate protected from light at 18°C–24°C for 5 min.
  d. Spin down the plate at 400 × *g* for 5 min at 18°C–24°C and carefully aspirate the supernatant.
  e. Repeat cell washes steps (5c and 5d) for a total of three times.
  f. Add 100 μL of previously prepared Assay Buffer (1×). Proceed immediately to measure the fluorescence intensity with a fluorescent plate reader using a filter designed to detect FITC (excitation/emission = 485/535 nm). Troubleshooting 2.

## Expected outcomes

The expected results of both assays are shown below (Figure 2). In Figure 2A, microglia are selected according to the following gating strategy: a) in a side scatter (SSC-A) - forward scatter (FSC-A) plot, brain cells are selected in order to exclude debris, dead cells and red blood cells; b) a FSC-H - FSC-A scatter plot allows the identification of single cells vs. doublets or multiple cells adhering to each other; c) signal from LIVE/DEAD Fixable Near-IR viability dye allows to identify dead cells that take up the dye vs. healthy cells that do not; d) microglia are identified as CD11b^high^ and CD45^int^ cells; e) translation rate is estimated through the detection of 5-Carboxyfluorescein (5-FAM) at the excitation wavelength of 485 nm and emission wavelength of 535 nm.

In Figure 2B, the plate reader relative fluorescent intensity (RFU) gives an estimate of translation rate in BMDMs.

In both cases, Cycloheximide treatment confirms that the detected fluorescence diminishes in the presence of a translational inhibitor. The extent of inhibition with CHX treatment is different between BMDMs and microglia and this might be due to different factors, such as incubation time with CHX, which is shorter for microglia. When designing the protocol for microglia, we aimed to reduce incubation times as much as possible, in order to reduce any potential loss of viability while microglia are in suspension. We did not aim to completely inhibit protein synthesis with our settings; it is possible that a higher concentration of CHX, in addition to longer incubation times, would completely inhibit protein synthesis, however, we have not tested them out of concern for microglia viability.

## Quantification and statistical analysis

Flow cytometry data analysis of translation rate in adult microglia can be carried out with FlowJo software (v10, BD Biosciences). A general guideline of data analysis is described below:

1. Run FlowJo (or alternative flow cytometry analysis software) on a computer.
2. Import .fcs files exported from the flow cytometer in FlowJo.
3. Apply compensation if needed.
4. Gate cells according to the gating strategy described in Figure 2A and identify microglia based on the expression of the CD11b and CD45 markers.
5. Determine the median fluorescent intensity (MFI) of 5-FAM signal within the CD11b^high^ CD45^int^ microglia population as a readout for translation rate.

Fluorescence intensity of protein synthesis in BMDMs can be easily determined by subtracting the blank (media only) from each condition in an excel file exported from a fluorescent plate reader. Statistical analysis and data visualization can be performed using GraphPad Prism.

## Limitations

The translation rate refers to the speed at which ribosomes translate the genetic information contained in mRNA molecules into proteins. This process can vary based on the cellular context and cell type being investigated. It is possible that the translation rate of microglia and BMDMs from certain genetic and/or disease models is too low to be detected and might be indistinguishable from the no OPP negative control. In this case, a possible solution might be to stimulate cells with growth factors that activate pathways promoting protein synthesis, such as the insulin growth factor-1 (IGF-1) for mTORC1-dependent translation.

Furthermore, in case it is not possible to use an adult brain cell suspension, neonatal primary microglia cultures could be used as an alternative, however, we have not previously tested it and some optimization might be required.

## Troubleshooting

### Problem 1

Cell-based assay fixative provided by the assay kit might lead to inconsistent results (flow cytometry-based translation assay in adult microglia – step 6; in-vitro translation assay for cultured bone marrow-derived macrophages (BMDMs) – step 3).

### Potential solution

We have encountered problems when using the assay fixative provided with the protein synthesis assay kit. This might be related to the formaldehyde solution not being stable when not freshly prepared. We solved this issue by replacing the assay fixative with a freshly prepared solution of 4% PFA in PBS (pH 7.4) that we routinely use for immunostaining assay.

### Problem 2

High fluorescent background in the “no OPP” control (flow cytometry-based translation assay in adult microglia – step 8f; in-vitro translation assay for cultured bone marrow-derived macrophages (BMDMs) – step 5f).

### Potential solution

The “no OPP’ condition is used as negative control for the assay, where cells are not treated with OPP but are subsequently treated with FAM-Azide. In this case, we expect a lower signal compared to CHX+OPP and OPP samples. In case the signal is higher, a possible explanation could be that the FAM-Azide has not been properly washed off the wells. If this occurs, we recommend increasing the number of washes, ensuring that cells are properly resuspended during the washing steps (for microglia) and that the supernatant is fully aspirated after centrifugation.

### Problem 3

Low cell viability in both assays (flow cytometry-based translation assay in adult microglia – step 2; in-vitro translation assay for cultured bone marrow-derived macrophages (BMDMs) – step 1).

### Potential solution

In both assays, the recommended centrifugation speed is the one indicated in the manufacturer’s protocol (400 × *g*). Although this speed is relatively high, we found that it did not affect the viability of microglia nor BMDMs. Nonetheless, if a decrease in cell viability is observed, it might be advisable to lower the centrifugation speed to 300 or even 200 × *g*, maintaining the same time of centrifugation (5 min). In this case, though, further care should be applied when aspirating supernatants.

### Problem 4

Myelin/lipids interfering with preparation of brain cell suspension, especially in aged brain (isolation of brain cells from adult mice – step 3).

### Potential solution

When using brains from aged mice, the increase in myelin/lipid content can compromise the preparation of the brain cell suspension. In this case, it might be necessary to use Myelin Removal beads (cat. no. 130-096-733, Miltenyi Biotec) after the debris removal step (step 3 for microglia in “isolation of brain cells from adult mice” paragraph), following the manufacturer’s guidelines.

### Problem 5

Incomplete dissociation of adult brain sample isolation of brain cells from adult mice – step 3).

### Potential solution

According to the manufacturer’s guideline of the Adult Brain Dissociation kit from Miltenyi Biotec, it is recommended to gently slice the brain in at least 8 sagittal pieces using a scalpel, in order to obtain a complete homogenization of brain tissue during the enzymatic digestion. If larger chunks are still visible after the enzymatic digestion, further homogenization of these pieces can be achieved by gently pipetting up and down with a P1000 tip before proceeding to the subsequent steps.

## Resource availability

### Lead contact

Further information and requests for resources and reagents should be directed to and will be fulfilled by the lead contact, Melania Capasso: melania.capasso@dzne.de.

### Materials availability

The study did not generate new unique reagents.

## Data Availability

This study has used data form the protein synthesis assay performed in BMDMs from Keane et al.^1^ The data from adult microglia have not been previously published. No bioinformatics code has been generated in this study.
